# Change in Patient Enrollment After Site Principal Investigator Turnover in Surgical Clinical Trials

**DOI:** 10.1001/jamanetworkopen.2024.15340

**Published:** 2024-06-06

**Authors:** Yao Tian, Charesa J. Smith, Salva Balbale, Willemijn Louise Albertine Schäfer, Jane L. Holl, Mehul V. Raval

**Affiliations:** 1 Department of Surgery, Feinberg School of Medicine, Northwestern University, Chicago, Illinois; 2 Division of Pediatric Surgery, Department of Surgery, Northwestern University Feinberg School of Medicine, Ann and Robert H. Lurie Children’s Hospital of Chicago, Chicago, Illinois; 3 Department of Gastroenterology and Hepatology, Feinberg School of Medicine, Northwestern University, Chicago, Illinois; 4 Center of Innovation for Complex Chronic Healthcare, Health Services Research and Development, Edward Hines, Jr. VA Hospital, Hines, Illinois; 5 Department of Neurology, Biological Sciences Division and Center for Healthcare Delivery Science and Innovation, University of Chicago, Chicago, Illinois

## Abstract

This cohort study examines the association of principal investigator (PI) turnover with patient enrollment in surgical clinical trials.

## Introduction

Stable patient enrollment is imperative to evaluate the implementation and effectiveness of an intervention in clinical trials. Previous studies have reported that successful patient enrollment is determined by the patient, physician, support staff, and institutional factors.^1^ Specifically, site principal investigators (PIs) typically play a central role in the process of patient enrollment.^2^ Over the course of a clinical trial, site PI turnover is a frequent occurrence.^3^ The purpose of this study was to evaluate patient enrollment after site PI turnover in the context of a randomized multicenter surgical clinical trial.

## Methods

In this cohort study, we conducted an interrupted time-series analysis to assess patient enrollment after site PI turnover, using data derived from an ongoing, prospective multicenter randomized clinical trial entitled Enhanced Recovery in Children Undergoing Surgery (ENRICH-US).^4^ The target population consists of pediatric patients, between 10 and 18 years of age, undergoing elective gastrointestinal surgery. Written informed consent was obtained from all participants who were at least 18 years old or their legal guardians if younger. The key variable of interest was a site PI turnover (ie, the prior PI left for another institution and handed off the study to another person) at each site, and the study outcome was the number of enrolled patients per quarter (collected from July 1, 2020, through August 31, 2023). The outcome was described using mean (SD) values, and a segmented regression was used to estimate the preturnover trend of patient enrollment, the immediate change of turnover, and the trend after turnover.^5^ A Durbin-Watson test was used to detect autocorrelation of residuals. All analyses were performed using SAS, version 9.4 (SAS Institute Inc). A significance level of .05 was used, and all tests were 2-tailed. The trial protocol was approved by Advarra, Inc (Columbia, Maryland), which serves as the central institutional review board for all study sites. This study followed the STROBE reporting guideline.

## Results

Over the study period, PI turnover occurred at 5 sites, where 158 eligible patients were enrolled (Table). Before the turnover, the mean (SD) number of enrolled patients per quarter was 3.2 (2.7). The mean (SD) number decreased to 1.0 (1.7) during the turnover quarter and was 1.3 (1.7) after the turnover. Results from the segmented regression, although not statistically significant, suggested a decreasing trend in patient enrollment before site PI turnover and an immediate decrease after turnover; the estimates for the preturnover trend, turnover quarter, and postturnover trend were −0.25 (*P* = .15), −1.37 (*P* = .26), and 0.39 (*P* = .29), respectively (Figure).

**Table.** Basic Demographic Information of Enrolled Patients^a^

Characteristic	Patients, No. (%)			*P* value
	Preturnover (n = 127)	Turnover quarter (n = 5)	Postturnover (n = 26)	
Age, median (IQR), y	15 (13-16)	15 (13-16)	15 (14-16)	.84
Sex				
Female	54 (42.5)	1 (20.0)	8 (30.8)	.39
Male	73 (57.5)	4 (80.0)	18 (69.2)	
Race and ethnicity				
Black	22 (17.3)	1 (20.0)	7 (26.92)	.15
Hispanic or Latino	23 (18.1)	0	0	
Non-Hispanic or Latino White	78 (61.4)	4 (80)	18 (69.2)	
No response^b^	4 (3.15)	0	1 (3.85)	

^a^ Over the study period, principal investigator turnover occurred at 5 sites (of a total of 18 sites), where 158 eligible patients were enrolled (of a total of 515 enrolled patients).

^b^ Participants did not indicate their races and ethnicities.

**Figure.**
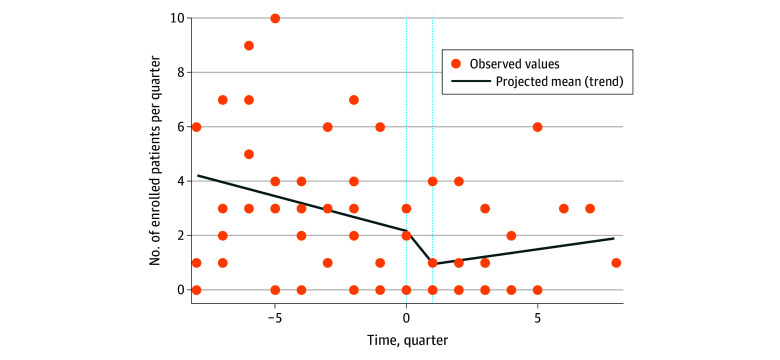
Estimated Preturnover Trend, Immediate Change, and Postturnover Trend of Patient Enrollment From the Segmented Regression The estimated changes in the number of enrolled patients per quarter per hospital for the preturnover trend, turnover quarter, and postturnover trend were −0.25 (*P* = .15), −1.37 (*P* = .26), and 0.39 (*P* = .29), respectively.

## Discussion

To our knowledge, this is the first study using quantitative data and methods to investigate patient enrollment after site PI turnover during a clinical trial. A decreasing trend in patient enrollment was noted leading up to site PI turnover and worsened abruptly at the time of site PI turnover. Enrollment gradually improved with time at most sites, after intensive orientation of a new site PI. The study has several limitations, including only 5 hospitals with a site PI turnover and a small sample of enrolled patients, thereby limiting statistical power. We did not conduct a comparative interrupted time-series analysis because of the low number of sites without a site PI turnover and the heterogeneity of sites. Nevertheless, the findings of this study can be used by investigators to tailor a strategic plan for future clinical trials. Specifically, efforts to facilitate site PI turnover, such as timely identification and adequate orientation of a new site PI, should be encouraged and prioritized to stabilize enrollment.

## Notes

Supplement.
Data Sharing Statement
