# Supplementary material for: Change in Patient Enrollment After Site Principal Investigator Turnover in Surgical Clinical Trials
Source: JAMA Netw Open. 2024 Jun 6;7(6):e2415340. doi: 10.1001/jamanetworkopen.2024.15340 (PMC11157349; doi:10.1001/jamanetworkopen.2024.15340)
Supplement: Supplement. — Data Sharing Statement [file jamanetwopen-e2415340-s001.pdf]

Data Sharing Statement

Tian. Change in Patient Enrollment After Site Principal Investigator Turnover in Surgical Clinical Trials. *JAMA Netw Open*. Published June 06, 2024.
doi:10.1001/jamanetworkopen.2024.15340

Data
Data available: No
